# N-3 polyunsaturated fatty acids improve lipoprotein particle size and concentration in Japanese patients with type 2 diabetes and hypertriglyceridemia: a pilot study

**DOI:** 10.1186/s12944-018-0706-8

**Published:** 2018-03-15

**Authors:** Kana Ide, Masaya Koshizaka, Hirotake Tokuyama, Takahiko Tokuyama, Takahiro Ishikawa, Yoshiro Maezawa, Minoru Takemoto, Koutaro Yokote

**Affiliations:** 1 Clinical Cell Biology and Medicine, Chiba University Graduate School of Medicine, Chiba, Japan; 2 Department of Diabetes, Metabolism and Endocrinology, Chiba University Hospital, Chiba, Japan; 3 National Hospital Organization Chiba Medical Center, Chiba, Japan; 4 Yu-karigaoka Tokuyama Clinic, Chiba, Japan; 5 Tokuyama Clinic, Chiba, Japan; 6 Department of Medicine, Division of Diabetes, Metabolism and Endocrinology, International University of Health and Welfare, Chiba, Japan

**Keywords:** Cardiovascular risk, Type 2 diabetes, Hypertriglyceridemia, N-3 polyunsaturated fatty acids, Lipoprotein, Small dense low-density lipoprotein cholesterol, Low-density lipoprotein cholesterol size, Dipeptidyl peptidase-4 inhibitor, Hydroxymethylglutaryl-CoA reductase inhibitor

## Abstract

**Background:**

Patients with type 2 diabetes are at high risk for cardiovascular disease. Although hydroxymethylglutaryl-CoA reductase inhibitors (statins) can reduce cardiovascular events, residual risk remains even after target low-density lipoprotein cholesterol (LDL-C) levels have been achieved. Lipoprotein particle size and fraction changes are thought to contribute to such risks. The purpose of this study was to evaluate the effects of n-3 polyunsaturated fatty acids (n-3 PUFAs), predominantly eicosapentaenoic acid and docosahexaenoic acid, on lipoprotein particle size, concentration, and glycemic control in Japanese patients with type 2 diabetes and hypertriglyceridemia.

**Methods:**

This was a multicenter, prospective, open-label, single arm study. We enrolled 14 patients with type 2 diabetes and hypertriglyceridemia treated with statins and dipeptidyl peptidase-4 inhibitors with glycated hemoglobin (HbA1c) < 8.0%, LDL-C < 120 mg/dL, and fasting triglyceride ≥150 mg/dL. After a 12-week observation period, they were treated with 4 g/day n-3 PUFAs for 12 weeks. Lipoprotein particle sizes, concentrations, lipoprotein insulin resistance (LPIR) scores, lipid profiles, HbA1c, and fasting plasma glucose (FPG) were measured before and after treatment. Lipoprotein profiles were measured by nuclear magnetic resonance spectroscopy. Data were analyzed using Wilcoxon signed-rank tests.

**Results:**

Concentrations of total cholesterol (*P* < 0.001), LDL-C (*P =* 0.003), and triglyceride (*P* < 0.001) decreased following n-3 PUFA administration. N-3 PUFAs decreased the size of very low-density lipoprotein (VLDL; *P* < 0.001) particles, but did not affect LDL or high-density lipoprotein (HDL) particles. The concentration of large LDL increased, whereas small LDL decreased, causing the large to small LDL ratio to increase significantly (*P* = 0.042). Large VLDL and chylomicron concentrations significantly decreased, as did the large to small VLDL ratio (all *P* < 0.001). FPG levels unchanged, whereas HbA1c levels slightly increased. LPIR scores improved significantly (*P =* 0.001).

**Conclusions:**

N-3 PUFAs partly improved atherogenic lipoprotein particle size and concentration, and produced less atherogenic lipoprotein subclass ratios in patients that achieved target LDL-C levels and glycemic control. These results suggest that n-3 PUFAs may reduce residual cardiovascular risk factors in statin-treated patients with type 2 diabetes and hypertriglyceridemia.

**Trial registration:**

The study was registered at UMIN-ID: UMIN000013776.

**Electronic supplementary material:**

The online version of this article (10.1186/s12944-018-0706-8) contains supplementary material, which is available to authorized users.

## Background

Over the past two decades, low-density lipoprotein cholesterol (LDL-C) has been established as a primary target for lipid-lowering therapies [1]. However, although numerous clinical trials have demonstrated that LDL-C-lowering therapy with hydroxymethylglutaryl-CoA reductase inhibitors (statins) can reduce cardiovascular (CV) events [2, 3], significant CV risks remain after achieving target LDL-C levels. Residual risk factors include hypertriglyceridemia, a low level of high-density lipoprotein (HDL) cholesterol, and increased small dense LDL or oxidized LDL.

Diabetes is also a strong risk factor for CV events. Insulin resistance (IR) frequently leads to high very low-density lipoprotein (VLDL) and low HDL concentrations; therefore, residual risks are often critical in patients with diabetes. In particular, hypertriglyceridemia is a potent risk factor for cardiovascular disease (CVD) in Japanese patients with type 2 diabetes [4]. However, the best strategy to target residual risk in patients with type 2 diabetes remains controversial. In the ACCORD lipid trial including 5518 patients with diabetes and high CV risk, the addition of fenofibrate [5] to simvastatin failed to reduce CV events, with significant risk reduction only in patients with hypertriglyceridemia and low HDL. In addition, fibrates often cause adverse effects when administered in combination with statins, especially in patients with renal dysfunction. The JELIS study [6] revealed that eicosapentaenoic acid (EPA) reduced residual CV risks, although no effects were observed in patients with dysglycemia [7]. Therefore, lipid-lowering therapies that can be used safely and effectively in statin-treated patients with diabetes are required.

In addition to lipoprotein concentrations, the importance of particle quality, such as size and function, has been recently considered. Small dense LDL particles are more atherogenic because of their lower binding affinity for the LDL receptor, longer plasma half-life, higher penetration into the arterial wall, and greater susceptibility to oxidative stress [8]. Previous studies have revealed that small dense LDL is strongly associated with CV events [9]. Furthermore, large VLDL is also considered atherogenic [10, 11].

Conversely, n-3 polyunsaturated fatty acids (n-3 PUFAs), predominantly EPA and docosahexaenoic acid (DHA), are widely used as a triglyceride (TG)-lowering therapy in patients with type 2 diabetes and hypertriglyceridemia [7]. EPA/DHA at 4 g/day, but not EPA at either 2 or 4 g/day [12], improved LDL particle size in patients with hypertriglyceridemia [13]. Moreover, a previous report revealed that the combination of n-3 PUFAs and dipeptidyl peptidase-4 (DPP-4) inhibitors was expected to improve glycemic control [14]. Therefore, n-3 PUFAs are expected to have multiple effects on the reduction of residual CV risks in statin-treated patients with type 2 diabetes.

Accordingly, the aim of this study was to evaluate the effects of n-3 PUFAs on residual CV risk factor reduction in a real-life setting. We assessed the effects of n-3 PUFAs on lipoprotein particle size, concentration, and glycemic control in Japanese patients with type 2 diabetes and hypertriglyceridemia who had achieved the target LDL-C level and glycemic control with statins and DPP-4 inhibitors.

## Methods

### Study design

This was a multicenter, prospective, open-label, single arm study. The study protocol was approved by the following Institutional Review Boards: Institutional Review Board of Chiba University Hospital (ID number: G25019) and National Hospital Organization Chiba Medical Center Research Review Board. Tokuyama Clinic was assessed at the Institutional Review Board of Chiba University Hospital, which was the centralized IRB (ID number: N25031). The protocol was registered at https://upload.umin.ac.jp/cgi-open-bin/ctr_e/ctr_view.cgi?recptno=R000016078 (UMIN-ID: UMIN000013776). Signed written informed consent was obtained from all patients. The study was conducted in full compliance with the articles of the Declaration of Helsinki.

### Patients

This study was conducted in Japanese patients with type 2 diabetes treated with statin and DPP-4 inhibitors, whose TG levels were not sufficiently controlled. The main inclusion criteria were: (1) treatment with statins and DPP-4 inhibitors, with an LDL-C level < 120 mg/dL (the target LDL-C level for Japanese patients with diabetes in primary prevention [15]), and a glycated hemoglobin (HbA1c) level < 8%; (2) fasting TG levels of ≥150 mg/dL twice, with at least 4 weeks between tests; and (3) age ≥ 20 years and < 80 years. The main exclusion criteria were: (1) TG level of ≥1000 mg/dL or HDL-C level of ≤30 mg/dL; (2) users of n-3 PUFAs, nicotinic acid, or fibrate; (3) pioglitazone use; (4) met the contraindication criteria of n-3 PUFAs; (5) hypersensitivity to EPA or DHA; (6) familial hypercholesterolemia; (7) diagnosed with type 1 diabetes; (8) recent history (within 6 months) of diabetic ketosis; (9) diagnosed with a severe infectious disease, was perioperative, or had experienced trauma; (10) serum creatinine level of ≥1.5 mg/dL (male) or 1.3 mg/dL (female); (11) recent history (within 6 months) of stroke or myocardial infarction, (12) diagnosed with cancer; or (13) pregnant, possibly pregnant, or lactating.

### Study procedures

After a 12-week observation period, patients received 12 weeks of treatment with n-3 PUFAs at 4 g/day (EPA 1860 mg and DHA 1500 mg; Lotriga^®^, Takeda Pharmaceutical Company Limited, Tokyo, Japan). Changes in lipid-altering agents were prohibited during the administration period. Antidiabetic medication could be changed, with the exception of pioglitazone. All patients in this study were educated regarding dietary and physical therapy by their doctor and dietitian when they were enrolled in the study. They continued the same diet and exercise during both the observation period and the intervention period. We verbally confirmed patient drug adherence every 4 weeks during the study period.

### Study endpoints

The primary endpoint was change in LDL particle size. The secondary endpoints were changes in lipoprotein particle size and concentration, lipoprotein insulin resistance (LPIR) score, which has strong associations with IR indices, glycoprotein acetylation (GlycA), which was an inflammatory marker, LDL-C, HDL-C, TG concentration, HbA1c, fasting plasma glucose (FPG), EPA/AA ratio, DHA/AA ratio, and safety value.

### Measurements

Overnight fasting blood samples were collected prior to and 4, 8, and 12 weeks after the administration of n-3PUFAs. Lipoprotein sizes and concentrations were measured prior to and 12 weeks after administration using the nuclear magnetic resonance (NMR)-based Lipoprofile-III^®^ method (Labcorp, Cary, NC) and a high performance liquid chromatography (HPLC)-based method (LipoSEARCH, Skylight Biotech, Tokyo, Japan).

### Statistical analysis

Data represent the means ± standard deviation (SD). Statistical analysis was performed using JMP Pro 12 (SAS, Cary, NC). A Wilcoxon signed-rank test was used for statistical analysis of clinical parameters and lipoprotein profiles prior to and following treatment. The Spearman rank correlation coefficient was used to analyze correlations between different variables. Differences were considered statistically significant when *P* < 0.05.

## Results

A total of 14 patients were enrolled in this study. Of these, two were withdrawn from the study, as one patient was treated with pioglitazone during the administration period, and one patient missed a fasting blood test. The rate of drug adherence exceeded 75%.

The baseline characteristics of the patients are shown in Table 1. The majority of patients achieved the targeted LDL-C level. However, their TG levels remained uncontrolled. There were no statistically significant changes during the observation period. Clinical parameters prior to and following treatment with n-3 PUFAs are presented in Table 2 and Additional file 1: Table S1. There were no significant differences in body mass index (BMI) or blood pressure before and after treatment. Fasting plasma glucose levels remained unchanged, whereas HbA1c levels increased (*P* = 0.038). Treatment with n-3 PUFAs significantly decreased the total cholesterol (*P* < 0.001), LDL-C (*P* = 0.003), and TG (*P* < 0.001) concentrations. The EPA/AA and DHA/AA ratios significantly increased (both *P* = 0.008) after treatment.

**Table 1:** Baseline characteristics

Characteristic	*n* = 12
Age (years)	65 ± 11
Male (%)	6 (50)
BMI (kg/m^2^)	28.2 ± 5.4
SBP (mmHg)	141 ± 7.6
DBP (mmHg)	74.9 ± 11.0
HbA1c (%)	6.9 ± 0.70
FPG (mg/dL)	139.5 ± 40.5
TC (mg/dL)	176.4 ± 26.8
LDL-C (mg/dL)	97.3 ± 25.8
HDL-C (mg/dL)	45.5 ± 9.1
TG (mg/dL)	184.7 ± 34.8
EPA/AA	0.30 ± 0.19
DHA/AA	0.75 ± 0.16
AST (IU/L)	24.5 ± 13.0
ALT (IU/L)	32.5 ± 31.7
Cre (mg/dL)	0.81 ± 0.30
Antidiabetic medications (%)	
DPP-4 inhibitor	12 (100)
Metformin	6 (50)
Glimepiride	3 (25)
Repaglinide	1 (8.3)
Miglitol	3 (25)
Ipragliflozin	1 (8.3)
Insulin Aspart	2 (16.6)
Insulin Detemir	1 (8.3)
Insulin Glargine	1 (8.3)
Hypolipidemic agents	
Rosuvastatin	6 (50)
Atorvastatin	2 (16.6)
Pravastatin	2 (16.6)
Pitavastatin	2 (16.6)
Antihypertensive agents	9 (75)
Microvascular complications	
Diabetes nephropathy	3 (25)
Macrovascular complications	
Cerebral infarction	1 (8.3)

Data represent the means ± standard deviation or n (%). *BMI* body mass index, *SBP* systolic blood pressure, *DBP* diastolic blood pressure, *HbA*1*c* glycated hemoglobin, *FPG* fasting plasma glucose, *TC* total cholesterol, *LDL-C* low-density lipoprotein cholesterol, *HDL-C* high-density lipoprotein cholesterol, *TG* triglyceride, *AST* aspartate aminotransferase, *ALT* alanine aminotransferase, *Cre* creatinine

**Table 2:** Changes in clinical parameters after administration of n-3 PUFAs

Parameter	Before	After	*P*-value
BMI (kg/m^2^)	28.2 ± 5.4	28.0 ± 5.4	0.700
SBP (mmHg)	141.0 ± 7.6	136.0 ± 11.3	0.113
DBP (mmHg)	74.9 ± 11.0	77.9 ± 11.0	0.334
HbA1c (%)	6.9 ± 0.5	7.1 ± 0.6	0.038
FPG (mg/dL)	132.3 ± 18.7	134.7 ± 25.8	0.664
TC (mg/dL)	171.3 ± 21.5	150.5 ± 16.0	< 0.001
LDL-C (mg/dL)	90.3 ± 17.1	79.4 ± 11.4	0.003
HDL-C (mg/dL)	44.9 ± 9.5	45.5 ± 11.3	0.763
TG (mg/dL)	195.7 ± 49.9	128.7 ± 58.8	< 0.001
EPA/AA	0.30 ± 0.19	1.30 ± 0.30	0.008
DHA/AA	0.75 ± 0.16	1.29 ± 0.32	0.008
AST (IU/L)	21.6 ± 6.0	21.8 ± 5.2	0.828
ALT (IU/L)	24.2 ± 6.0	25.4 ± 7.2	0.977
Cre (mg/dL)	0.81 ± 0.32	0.83 ± 0.36	0.539

Data represent the means ± standard deviation. *P*-values represent differences observed before and after administration of n-3 PUFAs. *BMI* body mass index, *SBP* systolic blood pressure, *DBP* diastolic blood pressure, *HbA1c* glycated hemoglobin, *FPG* fasting plasma glucose, *TC* total cholesterol, *LDL-C* low density lipoprotein cholesterol, *HDL-C* high density lipoprotein cholesterol, *TG* triglyceride, *AST* aspartate aminotransferase, *ALT* alanine aminotransferase, *Cre* creatinine

**Fig. 1:**
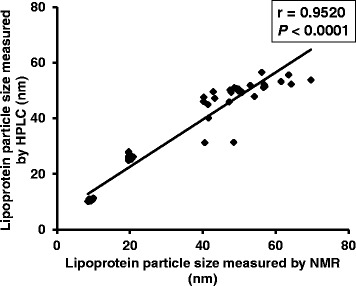
Lipoprotein particle size correlations using nuclear magnetic resonance (NMR) and high performance liquid chromatography (HPLC)

Table 3 displays the changes in lipoprotein particle size and concentration. Although LDL and HDL size remained unchanged, VLDL size significantly decreased (*P* < 0.001). Large VLDL and chylomicron concentrations significantly decreased (*P* < 0.001), and the small LDL concentration showed a tendency to decrease. Similarly, large VLDL and very small LDL particle numbers significantly decreased (*P* = 0.001 and *P* = 0.007, respectively; Additional file 2: Table S2). In addition, we confirmed a significant correlation between the NMR and HPLC methods with respect to lipoprotein particle size (Fig. 1).

**Table 3:** Changes in lipoprotein particles after n-3 PUFA administration measured by nuclear magnetic resonance

Lipoprotein particle	Before	After	*P*-value
VLDL size (nm)	56.3 ± 7.14	45.2 ± 4.01	< 0.001
LDL size (nm)	20.0 ± 0.306	20.1 ± 0.361	0.154
HDL size (nm)	9.13 ± 0.302	9.17 ± 0.382	0.535
Total VLDL & CM concentrations (nmol/L)	73.6 ± 22.0	75.6 ± 38.5	0.850
Large VLDL & CM (nmol/L)	10.4 ± 4.31	2.18 ± 1.81	< 0.001
Medium VLDL (nmol/L)	34.6 ± 11.2	36.5 ± 25.6	0.850
Small VLDL (nmol/L)	28.7 ± 15.3	36.8 ± 20.7	0.380
Total LDL concentration (nmol/L)	1040 ± 237	995 ± 215	0.266
IDL (nmol/L)	158 ± 75.8	155 ± 87.5	0.850
Large LDL (nmol/L)	98.9 ± 145	142 ± 131	0.142
Small LDL (nmol/L)	780 ± 210	698 ± 170	0.151
Total HDL concentration (nmol/L)	34.2 ± 4.42	32.0 ± 5.31	0.110
Large HDL (nmol/L)	6.25 ± 2.14	6.74 ± 3.26	0.531
Medium HDL (nmol/L)	4.73 ± 5.00	3.93 ± 3.60	0.557
Small HDL (nmol/L)	23.2 ± 4.71	21.3 ± 3.42	0.233
LPIR score	65.1 ± 11.1	40.5 ± 14.8	0.001
GlycA	411 ± 50.5	432 ± 49.1	0.129

Data represent the means ± standard deviation. *P*-values represent differences observed before and after n-3 PUFA administration. *VLDL* very low-density lipoprotein, *LDL* low-density lipoprotein, *HDL* high-density lipoprotein, *CM* chylomicron, *IDL* intermediate density lipoprotein, *LPIR* lipoprotein insulin resistance, *GlycA* glycoprotein acetylation

**Fig. 2:**
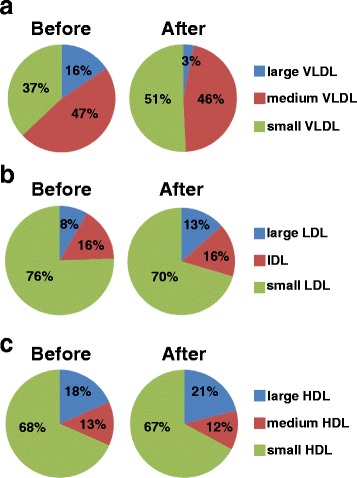
Changes in lipoprotein subclass ratios after n-3 PUFA treatment. Panels show the subclass percentages for (**a**) very low-density lipoprotein (VLDL), (**b**) low-density lipoprotein (LDL), and (**c**) high-density lipoprotein (HDL). IDL, intermediate density lipoprotein

Figure 2 displays the changes in the lipoprotein subclass ratios. After treatment, the percentage of large VLDL decreased from 15.6% to 3.2% whereas small VLDL increased from 37.0% to 50.7% (Fig. 2a). The ratio of large to small VLDL significantly decreased from 0.79 to 0.09 (*P* < 0.001). Large LDL increased from 8.4% to 13.5% whereas small LDL decreased from 75.6% to 70.5% (Fig. 2b). The ratio of large to small LDL significantly increased from 0.14 to 0.21 (*P* = 0.042). Large HDL increased from 18.4% to 21.0% whereas medium HDL decreased from 13.2% to 11.8% (Fig. 2c). The ratio of large to medium HDL tended to increase, from 7.5 to 9.1 (*P* = 0.79). In addition, LPIR scores significantly improved (*P =* 0.001) after treatment with n-3 PUFAs whereas GlycA remained unchanged (*P* = 0.129; Table 3).

There were no severe adverse events related to treatment, and n-3 PUFAs had no effect on liver or renal function (Table 2). One patient complained of mild constipation and another of a symptom similar to hypoglycemia. Both conditions were considered to be unrelated to treatment.

**Fig. 3:**
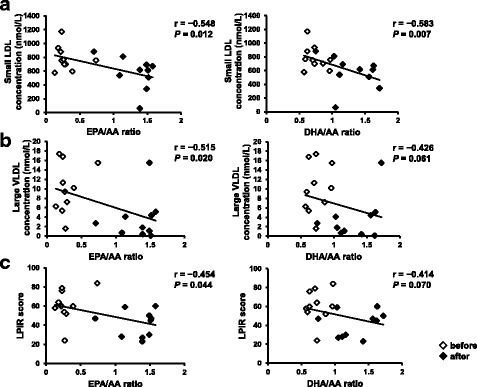
Correlations between the EPA/AA and DHA/AA ratios and (**a**) small LDL concentration, (**b**) large VLDL concentration, (**c**) lipoprotein insulin resistance (LPIR) score. LDL, low-density lipoprotein; VLDL, very low-density lipoprotein

Figure 3 displays the correlations between the EPA/AA and DHA/AA ratios and the lipoprotein particle concentrations and LPIR scores. The small LDL concentration was significantly negatively correlated with the EPA/AA (*r* = − 0.548, *P* = 0.012) and DHA/AA (*r* = − 0.583, *P* = 0.007) ratios (Fig. 3a). Both the large VLDL concentration and LPIR score were significantly negatively correlated with the EPA/AA ratio (*r* = − 0.515, *P* = 0.020 and *r* = − 0.454, *P* = 0.044, respectively; Fig. 3b and c). Table 4 displays the baseline clinical parameters in the subgroups exhibiting decreased and increased very small LDL particle concentrations. In the former, the baseline TG level was significantly higher (*P* = 0.026). Additional file 3: Figure S1 shows the relationship between HbA1c and DHA/AA or EPA/AA ratio. Notably, change of HbA1c was positively correlated with change of DHA/AA (*r* = 0.779, *P* = 0.023) but not EPA/AA ratio.

**Table 4:** Baseline clinical parameters in very small LDL particle subgroups with decreased and increased concentration

Parameter	Very small LDL particle concentration		
	Patients with decrease (*n* = 9)	Patients with increase (*n* = 3)	*P*-value
Age (years)	62.6 ± 11.1	72.0 ± 4.00	0.194
BMI (kg/m^2^)	28.4 ± 5.34	27.9 ± 6.89	1.000
TC (mg/dL)	176 ± 26.8	178 ± 32.7	1.000
LDL-C (mg/dL)	95.8 ± 26.1	102 ± 30.0	1.000
HDL-C (mg/dL)	44.1 ± 8.12	50.0 ± 12.7	0.350
TG (mg/dL)	195 ± 34.6	154 ± 5.86	0.026

Data represent the means ± standard deviation. *P*-values represent differences between the two groups. Lipoprotein particle concentrations were measured using high performance liquid chromatography. *BMI* body mass index, *TC* total cholesterol, *LDL-C* low-density lipoprotein cholesterol, *HDL-C* high-density lipoprotein cholesterol, *TG* triglyceride

## Discussion

In this study, we found that n-3 PUFAs could reduce the ratio of atherogenic lipoprotein particles and reduce the LPIR score in patients with type 2 diabetes, whereas there were no marked changes in LDL particle size and concentration. These findings are clinically important in two respects: (1) n-3 PUFAs may safely reduce residual CV risks in patients with type 2 diabetes who were treated with statins and achieved target LDL-C levels, and (2) n-3 PUFAs might reduce lipoproteins associated with IR in patients with type 2 diabetes.

There are few studies that address patients who have achieved LDL-C target levels through statin use, although some studies have demonstrated that statins and n-3 PUFAs improved lipoprotein particle size and number [16–18]. Dunbar et al. [19] reported that 6 weeks of treatment with 4 g/day n-3 PUFAs reduced atherogenic lipoprotein particle concentrations and increased LDL size in patients treated with statins. Their results indicated that changes in LDL particle size were correlated with changes in the TG level. Conversely, in our study, n-3 PUFAs did not improve LDL particle size. A potential explanation for this difference is that primary TG levels in this study (≥150 mg/dL) were not as high as in the previous study (≥200 mg/dL). However, small LDL particles were significantly decreased in patients with primary high TG in our study. These results thus support the conclusion that the TG level mainly determines LDL particle size [20].

Some studies have reported increased LDL-C concentrations after n-3 PUFA therapy in patients with hypertriglyceridemia [21], because n-3 PUFAs form smaller VLDL particles, which are more rapidly converted to LDL than large VLDL particles [22]. In contrast, the LDL-C concentration did not increase after treatment in our study. A possible explanation for this result may be that statin coadministration activated LDL receptors, which led to enhanced clearance of intermediate density lipoprotein and LDL [23]. Furthermore, the LDL-C level is correlated with the TG level. When TG ≥ 133 mg/dL, LDL-C levels increase as TG levels decrease; however, in the range of TG < 133 mg/dL, LDL-C levels decrease as TG levels decrease [24]. In our study, the TG level markedly decreased (from 195.7 ± 49.9 to 128.7 ± 58.8 mg/dL), which may explain why the LDL-C concentration did not increase.

N-3 PUFAs significantly decreased VLDL particle size and concentration, especially for large VLDL. In patients with type 2 diabetes, hypertriglyceridemia causes both overproduction of TG-rich VLDL and impaired VLDL clearance [25]. TG-rich VLDL is larger, and can promote endothelial dysfunction by inhibiting endothelium-dependent vasorelaxation [10, 11]. In addition, increased large VLDL promotes cholesteryl ester transfer protein-catalyzed TG exchange from VLDL to LDL. TG-rich LDL is a substrate for hepatic lipase, resulting in the formation of small dense LDL. For this reason, large VLDL is atherogenic, and reduced large VLDL is considered an anti-atherogenic change.

The LPIR score constitutes a novel marker associated with IR that comprises six lipoprotein parameters (VLDL, LDL, and HDL particle sizes and large VLDL, small LDL, and large HDL particle concentrations) as measured by NMR. The LPIR score was strongly correlated with the homeostasis model assessment of IR (HOMA-IR) and glucose disposal rate in nondiabetic patients [26]. Supplementation of n-3 PUFAs improved IR in an animal model, as measured by the HOMA-IR [27]. In the present study, the LPIR score significantly decreased after administration of n-3 PUFAs, indicating that n-3 PUFAs can decrease IR-related lipoprotein particles. However, a limitation of our study is that we did not examine other IR measures, such as the HOMA-IR.

The effect of n-3 PUFAs on glycemic control in patients with type 2 diabetes is controversial [28–30]. Iwasaki et al. [14] reported that reduction of HbA1c by DPP-4 inhibitors significantly correlates with serum EPA and DHA levels; therefore, DPP-4 inhibitors and n-3 PUFAs were expected to be a good combination. However, in our study, the HbA1c level slightly increased during the treatment period. This may have occurred in part because of the short treatment duration and use of statins. Furthermore, unlike a previous report [14], patients in the present study were also treated with other anti-diabetic medications, reflecting the clinical settings. These factors are thought to deteriorate glycemic control. In addition, our study revealed that the increase in HbA1c was positively correlated with increase in DHA/AA. Contrary to previous studies, DHA might thus increase HbA1c in some situations; therefore, care should be taken regarding the deterioration of glycemic control in statin-treated patients with diabetes.

This study has several limitations. First, this was an open-label and single-arm design study. Therefore, a randomized controlled study is required. However, the present study showed that n-3 PUFAs changed the ratio of lipoprotein particles in favor of less atherogenic particles. Moreover, the EPA/AA and DHA/AA plasma ratios were significantly negatively correlated with atherogenic particle concentrations. There were also no statistically significant changes in clinical parameters during the observation period. For these reasons, we consider that these effects were not the result of diet or other medications, but arose following the administration of n-3 PUFAs. Second, the small number of patients may be insufficient to evaluate changes in lipoprotein particles. However, this was the first study to evaluate the effects of DPP-4 inhibitor, statin, and n-3 PUFA combination therapy on lipoprotein particle and glycemic control in patients with type 2 diabetes and hypertriglyceridemia. Larger studies will be required in the future to confirm our findings. Third, we did not take into account the effect of diet or nutraceuticals on lipoprotein profiles. Recent reports have indicated that several nutraceuticals have lipid-lowering effects [31–33] and are associated with the risk of arteriosclerotic disease [34]. In this study, all patients were educated regarding diet therapy; however, we could not collect actual data related to diet and use of nutraceuticals. Therefore, we cannot completely exclude the effects of dietary factors on our results. Lastly, we did not evaluate the effects of n-3 PUFAs on the development of arteriosclerotic diseases directly, owing to the short treatment duration.

## Conclusions

N-3 PUFAs improved atherogenic lipoprotein particle size and concentration. This treatment changed the ratio of lipoprotein subclasses to less atherogenic values, even in patients that had achieved target LDL-C levels and glycemic control. N-3 PUFAs may reduce residual CV risk factors in patients with type 2 diabetes and hypertriglyceridemia.

## Additional files

Additional file 1: **Table S1.** Changes in clinical parameters 4 and 8 weeks after the administration of n-3 polyunsaturated fatty acids (n-3 PUFAs) (DOCX 22 kb)

Additional file 2: **Table S2.** Changes in lipoprotein particles after n-3 polyunsaturated fatty acid (n-3 PUFA) administration measured by high performance liquid chromatography (DOCX 22 kb)

Additional file 3: **Figure S1.** Correlations between ΔHbA1c and the ratios of ΔDHA/AA and ΔEPA/AA. Data present the changes in HbA1c, DHA/AA ratio, and EPA/AA ratio from baseline to the end of the intervention period (PDF 56 kb)

## Abbreviations

CV: Cardiovascular
CVD: Cardiovascular disease
DHA: Docosahexaenoic acid
DPP-4: Dipeptidyl peptidase-4
EPA: Eicosapentaenoic acid
FPG: Fasting plasma glucose
Glyc-A: Glycoprotein acetylation
HbA1c: Glycated hemoglobin
HDL: High-density lipoprotein
HOMA-IR: Homeostasis model assessment of insulin resistance
HPLC: High performance liquid chromatography
IR: Insulin resistance
LDL-C: Low-density lipoprotein cholesterol
LPIR: Lipoprotein insulin resistance
n-3 PUFAs: n-3 polyunsaturated fatty acids
NMR: Nuclear magnetic resonance
TG: Triglyceride
VLDL: Very low-density lipoprotein
