# Supplementary material for: N-3 polyunsaturated fatty acids improve lipoprotein particle size and concentration in Japanese patients with type 2 diabetes and hypertriglyceridemia: a pilot study
Source: Lipids Health Dis. 2018 Mar 15;17:51. doi: 10.1186/s12944-018-0706-8 (PMC5855932; doi:10.1186/s12944-018-0706-8)
Supplement: Supplementary file 1 — Table S1. Changes in clinical parameters 4 and 8 weeks after the administration of n-3 polyunsaturated fatty acids (n-3 PUFAs) (DOCX 22 kb) [file 12944_2018_706_MOESM1_ESM.docx]

**Table S1.** Changes in clinical parameters 4 and 8 weeks after the administration of n-3 polyunsaturated fatty acids (n-3 PUFAs)

| Parameter | 4 weeks | *P*-value | 8 weeks | *P*-value |
| --- | --- | --- | --- | --- |
| HbA1c (%) | 6.9 ± 0.5 | 0.766 | 6.9 ± 0.5 | 0.328 |
| FPG (mg/dL) | 137.5 ± 20.2 | 0.232 | 132.9 ± 18.8 | 0.904 |
| TC (mg/dL) | 151.2 ± 22.0 | 0.002 | 154.6 ± 17.0 | 0.001 |
| LDL-C (mg/dL) | 76.3 ± 19.3 | 0.002 | 82.6 ± 14.9 | 0.064 |
| HDL-C (mg/dL) | 45.5 ± 12.7 | 0.528 | 44.0 ± 8.2 | 0.454 |
| TG (mg/dL) | 151.4 ± 58.9 | 0.005 | 135.4 ± 45.5 | 0.003 |
| AST (IU/L) | 22.8 ± 8.8 | 1.000 | 27.1 ± 10.2 | 0.078 |
| ALT (IU/L) | 27.7 ± 9.2 | 0.102 | 27.6 ± 9.4 | 0.551 |
| Cre (mg/dL) | 0.83 ± 0.35 | 0.640 | 0.85 ± 0.44 | 0.190 |

Data represent the mean ± standard deviation. *P*-values represent differences observed before and 4 or 8 weeks after n-3 PUFA administration. HbA1c, glycated hemoglobin; FPG, fasting plasma glucose; TC, total cholesterol; LDL-C, low density lipoprotein cholesterol; HDL-C, high density lipoprotein cholesterol; TG, triglyceride; AST, aspartate aminotransferase; ALT, alanine aminotransferase; Cre, creatinine.
