# Supplementary material for: N-3 polyunsaturated fatty acids improve lipoprotein particle size and concentration in Japanese patients with type 2 diabetes and hypertriglyceridemia: a pilot study
Source: Lipids Health Dis. 2018 Mar 15;17:51. doi: 10.1186/s12944-018-0706-8 (PMC5855932; doi:10.1186/s12944-018-0706-8)
Supplement: Supplementary file 2 — Table S2. Changes in lipoprotein particles after n-3 polyunsaturated fatty acid (n-3 PUFA) administration measured by high performance liquid chromatography (DOCX 22 kb) [file 12944_2018_706_MOESM2_ESM.docx]

**Table S2.** Changes in lipoprotein particles after n-3 polyunsaturated fatty acid (n-3 PUFA) administration measured by high performance liquid chromatography

| Lipoprotein particle | Before | After | *P*-value |
| --- | --- | --- | --- |
| VLDL particle size (nm) | 51.1 ± 2.21 | 47.4 ± 3.39 | 0.004 |
| LDL particle size (nm) | 25.7 ± 0.790 | 25.9 ± 0.686 | 0.079 |
| HDL particle size (nm) | 10.4 ± 0.173 | 10.5 ± 0.304 | 0.065 |
| CM particle number (nmol/L) | 2.27 ± 1.43 | 0.820 ± 0.620 | <0.001 |
| Total VLDL particle number (nmol/L) | 168 ± 36.2 | 138 ± 56.3 | 0.012 |
| Large VLDL (nmol/L) | 73.7 ± 14.2 | 50.7 ± 24.7 | 0.001 |
| Medium VLDL (nmol/L) | 54.9 ± 14.2 | 48.4 ± 22.3 | 0.110 |
| Small VLDL (nmol/L) | 39.1 ± 12.2 | 38.8 ± 12.3 | 1.000 |
| Total LDL particle number (nmol/L) | 1080 ± 213 | 960 ± 167 | 0.003 |
| Large LDL (nmol/L) | 148 ± 26.7 | 148 ± 23.4 | 1.000 |
| Medium LDL (nmol/L) | 441 ± 97.8 | 396 ± 70.4 | 0.002 |
| Small LDL (nmol/L) | 306 ± 78.4 | 259 ± 64.4 | 0.005 |
| Very small LDL (nmol/L) | 182 ± 35.2 | 157 ± 38.7 | 0.007 |
| Total HDL particle number (nmol/L) | 14600 ± 1670 | 13700 ± 1690 | 0.007 |
| Very large HDL (nmol/L) | 185 ± 50.6 | 190 ± 63.3 | 0.519 |
| Large HDL (nmol/L) | 828 ± 381 | 1020 ± 548 | 0.034 |
| Medium HDL (nmol/L) | 3130 ± 631 | 2920 ± 760 | 0.016 |
| Small HDL (nmol/L) | 5370 ± 486 | 4770 ± 591 | <0.001 |
| Very small HDL (nmol/L) | 5080 ± 552 | 4830 ± 605 | 0.176 |

Data represent the mean ± standard deviation. *P*-values represent differences observed before and after administration of n-3 PUFAs. VLDL, very low-density lipoprotein; LDL, low-density lipoprotein; HDL, high-density lipoprotein; CM, chylomicron.
