# Supplementary figures and images for: N-3 polyunsaturated fatty acids improve lipoprotein particle size and concentration in Japanese patients with type 2 diabetes and hypertriglyceridemia: a pilot study
Source: Lipids Health Dis. 2018 Mar 15;17:51. doi: 10.1186/s12944-018-0706-8 (PMC5855932; doi:10.1186/s12944-018-0706-8)

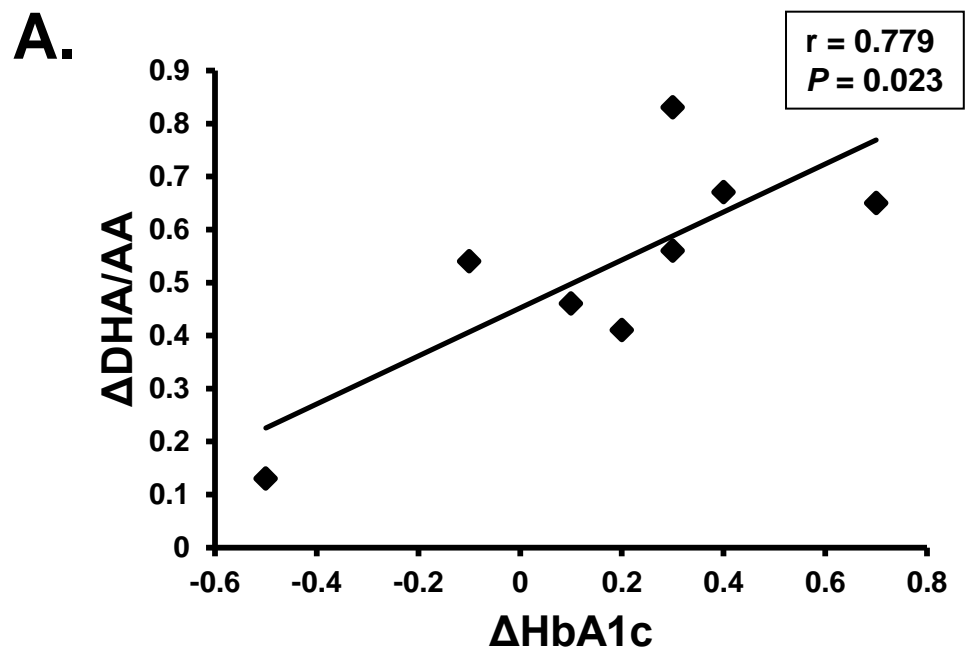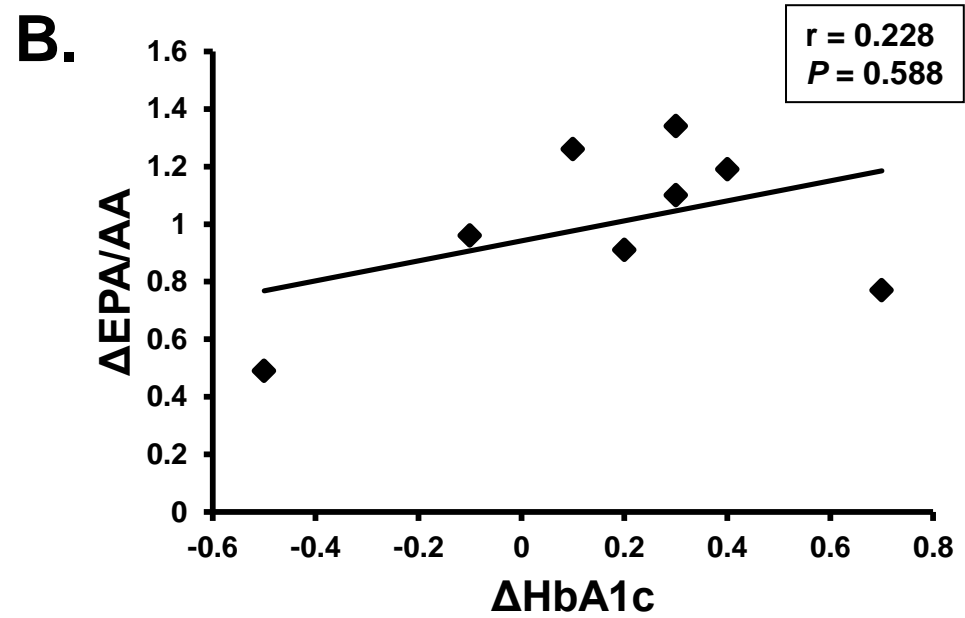

Supplement: Supplementary file 3 — Figure S1. Correlations between ΔHbA1c and the ratios of ΔDHA/AA and ΔEPA/AA. Data present the changes in HbA1c, DHA/AA ratio, and EPA/AA ratio from baseline to the end of the intervention period (PDF 56 kb) [file 12944_2018_706_MOESM3_ESM.pdf]
